# Addressing the risk of inadequate and excessive micronutrient intakes: traditional versus new approaches to setting adequate and safe micronutrient levels in foods

**DOI:** 10.3402/fnr.v58.26020

**Published:** 2015-01-27

**Authors:** Maaike J. Bruins, Gladys Mugambi, Janneke Verkaik-Kloosterman, Jeljer Hoekstra, Klaus Kraemer, Saskia Osendarp, Alida Melse-Boonstra, Alison M. Gallagher, Hans Verhagen

**Affiliations:** 1 DSM Biotechnology Center, Delft, The Netherlands; 2 Nutrition and Dietetics Unit, Ministry of Health, Nairobi, Kenya; 3 National Institute for Public Health and the Environment (RIVM), Bilthoven, The Netherlands; 4 Sight and Life, Basel, Switzerland; 5 Johns Hopkins Bloomberg School of Public Health, Baltimore, MD, USA; 6 The Micronutrient Initiative, Ottawa, Canada; 7 Division of Human Nutrition, Wageningen University, Wageningen, The Netherlands; 8 Northern Ireland Centre for Food and Health, University of Ulster, Coleraine, Northern Ireland

**Keywords:** food fortification, nutrient reference values, requirements, cut-point method, risk–benefit assessment, public health

## Abstract

Fortification of foods consumed by the general population or specific food products or supplements designed to be consumed by vulnerable target groups is amongst the strategies in developing countries to address micronutrient deficiencies. Any strategy aimed at dietary change needs careful consideration, ensuring the needs of at-risk subgroups are met whilst ensuring safety within the general population. This paper reviews the key principles of two main assessment approaches that may assist developing countries in deciding on effective and safe micronutrient levels in foods or special products designed to address micronutrient deficiencies, that is, the cut-point method and the stepwise approach to risk–benefit assessment. In the first approach, the goal is to shift population intake distributions such that intake prevalences below the Estimated Average Requirement (EAR) and above the Tolerable Upper Intake Level (UL) are both minimized. However, for some micronutrients like vitamin A and zinc, a narrow margin between the EAR and UL exists. Increasing their intakes through mass fortification may pose a dilemma; not permitting the UL to be exceeded provides assurance about the safety within the population but can potentially leave a proportion of the target population with unmet needs, or *vice versa*. Risk–benefit approaches assist in decision making at different micronutrient intake scenarios by balancing the magnitude of potential health benefits of reducing inadequate intakes against health risks of excessive intakes. Risk–benefit approaches consider different aspects of health risk including severity and number of people affected. This approach reduces the uncertainty for policy makers as compared to classic cut-point methods.

Micronutrient malnutrition or ‘hidden hunger’ is a global health problem affecting 2 billion people primarily in low-income countries (1). Young children and pregnant and breastfeeding women are most vulnerable to micronutrient deficiency due to their relative high requirements for growth, development, and reproduction (2). Micronutrient deficiency causes impaired immune and visual function, poor physical, and cognitive development as well as increased risk for anemia and mortality (3–5). Worldwide, childhood vitamin A and zinc deficiency account for 9% of the childhood burden of morbidity and mortality, followed by iodine and iron accounting for another 0.2% (5). Both clinical and subclinical forms of micronutrient deficiencies contribute considerably to the global disease and economic burden (6).

Micronutrient deficiencies have multiple causes, and therefore, there is no single strategy to eliminate micronutrient deficiencies suitable for all situations (3). A combination of programs such as promotion of breastfeeding, education, and control of infectious diseases alongside micronutrient interventions is essential to tackle malnutrition (3). Key interventions to combat micronutrient malnutrition at the level of populations or groups include dietary diversification, biofortification, food fortification, use of special nutritional products, and high-dose supplements (3, 7). More recently, evidence for the effectiveness of large-scale micronutrient interventions in reducing the global disease burden has increased significantly (4).

Fortification can be a high-priority investment if widespread deficiencies exist (8). The choice between mandatory or voluntary food fortification usually depends on national circumstances (9). Mass food fortification aims to reach the majority of the population and is generally the best approach when the majority of the population has an unacceptable health risk of micronutrient deficiency (9). It requires that a centrally processed basic commodity is available that is fortifiable at a reasonable cost, and is consumed in constant quantities by the general population (9). To date salt iodization, flour fortification with folic acid, and sugar/oil fortification with vitamin A are amongst the most cost-effective fortification strategies (8, 10, 11). In most developing countries, foods are fortified by law, but the contribution of voluntary food fortification to micronutrient intake is expanding as fortified processed foods gain both popularity and market in developing countries, particularly within urban areas (9).

Biofortification, which is the purposive breeding of (staple) crops with a higher micronutrient content (both via genetic engineering and conventional breeding strategies), has recently been developed as a new strategy to combat micronutrient deficiencies (12). Crops that are currently under development are, for example, beans with increased iron content; pearl millet, rice, and wheat with increased zinc content; and yellow maize and cassava with increased pro-vitamin A content (12). Promising results from efficacy studies are currently boosting the success of this strategy, and several biofortified crops have already been released in developing countries.

If mass fortification fails to reach the sub-population at greatest risk of micronutrient deficiency (typically children under five or pregnant and breastfeeding women), then targeted approaches are likely to have more potential (9). One form of targeted delivery of micronutrients is the periodical delivery of high-dose single micronutrient supplements in the form of tablets, capsules, or syrup often through national health campaigns. Successful supplementation programs for young children include vitamin A supplements every 4–6 months to reduce all-cause mortality in children 6–59 months, zinc supplements for 10–14 days to reduce diarrhea in children, and intermittent use of iron supplements to prevent or treat iron deficiency anemia in preschool or school-age children (13). Other targeted strategies include delivery of special nutritional products intended to deliver multiple micronutrients with the food on a daily base to particular at-risk groups. A number of special nutritional products have been developed to combat malnutrition in developing countries such as cereal-based fortified blended foods, high-energy biscuits, ready-to-use therapeutic foods, and complementary food supplements, such as lipid-based nutrient supplements and micronutrient powders (7, 14). Targeted delivery of micronutrients has the advantage that it can reach the target population without risk of excessive intakes to the wider population (15).

In industrialized countries, safety is often regulated at the high end of intake due to main concern of excessive intakes from widespread voluntary use of dietary supplements and extensive voluntary fortification of foods. In contrast, in countries with widespread micronutrient deficiencies, adverse health risks of micronutrient deficiency are the main public health concern. Nevertheless, some developing countries face concern of micronutrient deficiencies and also of potentially excessive intakes due to the expansion of multiple concurrent efforts to raise micronutrient intakes (i.e. high-dose supplements, fortified foods, special nutritional products). This concern may be particularly relevant if the additional levels to foods or food supplements are uncontrolled or if consumption of the food is unevenly distributed within the population. Therefore, regulation of minimum micronutrient addition levels is needed to ensure that the intended amount of micronutrients reaches the target population whilst ensuring that the maximum levels of intake from all sources are safe for the general population (9).

In this paper, two main conceptual approaches are discussed that can be used to estimate effective and safe micronutrient additions to foods used in programs to control micronutrient malnutrition; the traditional cut-point method; and the newer stepwise approach to risk–benefit assessments.

## Methods using intake distribution and EAR and UL as cut-points

Traditional methods employed in planning micronutrient intakes for population age and gender groups are based on usual intake distributions of the micronutrient in relation to its reference values. The goal is to plan for usual micronutrient intakes that have an acceptably low probability of inadequacy or excess. The Estimated Average Requirement (EAR) and Tolerable Upper Intake Level (UL) are usually taken as a cut-point to shift the intake distribution (9, 16, 17); intakes below the EAR are thought to reflect risks of inadequacy and intakes above the UL to reflect risks of excess. The recommended planning strategy of methods using the EAR as cut-point is to shift the micronutrient intake distribution upwards such that only 2.5% of the target population has intakes below the EAR, that is, considered at risk of inadequate intakes (9, 16). Meanwhile, the UL is often taken as cut-point on the upper end of intake distribution to minimize the proportion of the population with too high intakes (9). The Food and Nutrition Board of the US Institute of Medicine has described the principles of the EAR cut-point method (16) and the World Health Organization (WHO)/Food and Agriculture Organization of the United Nations (FAO) described in detail how to apply the method to set fortification levels (9).

### What do the EAR and UL cut-points reflect in terms of risk

**Fig. 1:**
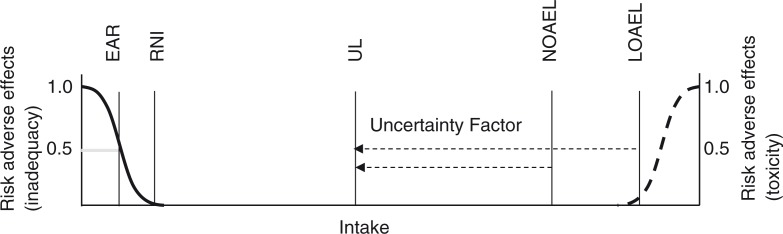
The risks of adverse health effects from decreasing intakes and the risks of adverse health effects with increasing intakes. The Estimated Average Requirement (EAR) reflects the intake where 50% of a population group is at risk of inadequacy, whereas the Tolerable Upper Intake Level (UL) is set an uncertainty factor lower than the No Observed Adverse Effect Level (NOAEL) or Lowest Observed Adverse Effect Level (LOAEL). The Recommended Nutrient Intake (RNI) is set at two standard deviations above the EAR and reflects the intake level at which 2.5% of a population group is at risk of inadequacy.

Usual intakes at the EAR represent intakes that are inadequate for the prevention of inadequacy-related adverse effects in 50% of the population (18, 19). The EAR values are established based on adverse effect indicators of inadequacy described in the scientific literature as, for example, serum or urinary biochemical markers, clinical markers, or intakes needed to maintain normal plasma ranges (18, 19). The Recommended Nutrient Intake (RNI) is set at two standard deviations above the EAR. Similarly, literature-reported biochemical or clinical adverse effect indicators of excessive micronutrient intake, if any, are used to establish the highest intake of a nutrient at which adverse effects have not been observed, No Observed Adverse Effect Level (NOAEL), or the lowest intake at which a relevant adverse effect has been demonstrated, Lowest Observed Adverse Effect Level (LOAEL) (18–20). Whereas the intake dose–adverse effect relationship is more or less known for most micronutrients at the low end of the intake spectrum, very little is known for most micronutrients at the high end of the intake spectrum (20, 21). To deal with the uncertainty around the established NOAEL (or LOAEL), an uncertainty factor is applied to reduce the level to a safe UL; the larger the uncertainty, the more conservative the UL (20, 21). This uncertainty factor takes into account uncertainty including extrapolation from animals to humans, from sub-chronic to chronic exposure, and across age groups. Usual intakes at the UL therefore represent the highest level of intake that is likely to pose no risks of adverse effects in an age and gender group (18, 19). As such, risks associated with intakes below the EAR and above the UL are different (18, 19). The EAR is the midpoint of required intakes at which the risk of inadequacy is 50%, whereas close to the UL the risk is negligible as the UL is set an uncertainty factor lower than the intake levels at and above which adverse effects may be expected (Fig. 1).

### Requirements and programs for the EAR (and UL) cut-point method

To evaluate the pre-intervention situation and simulate the post-intervention situation, food intake data are required. Food consumption surveys provide insight into the intake distribution of foods and micronutrients in a population and assist in identifying micronutrient deficiencies of concern to be addressed, the groups at risk, and the most suitable food vehicles for fortification (22).

The EAR cut-point method requires the distribution of micronutrient requirements to be symmetrical, the variance of the requirements to be less than the variance of the usual intake distribution, and intakes and requirements to be independent (23). For example, iron requirement distribution in menstruating women is asymmetrical, necessitating a probability approach instead of the EAR cut-point method (24). The EAR cut-point method also performs less well when the true prevalence of inadequacy in the group is very small or very large (23).

Recently, software has been developed that can simulate distributions of usual intake of foods and nutrients based on consumption data (Intake Monitoring, Assessment and Planning Program, IMAPP) (25). The program can also simulate how different food vehicles and micronutrient addition levels would change the prevalence of micronutrient intakes below the EAR and above the UL in different population groups (25). The user can select the form and the bioavailability (if different from default) of the nutrient. This can assist in selecting the most suitable food vehicle and fortification level. Other programs have been developed that can predict intake distributions from the usual diet and from additional intake, which may be particularly useful for micronutrients with a skewed intake distribution (26). However, few low- and middle-income countries have (reliable) food intake data available, and simulation of food intake is rarely undertaken. Countries that successfully applied the EAR cut-point method using food intake data to identify the most common micronutrient deficiencies and most suitable food vehicles include the Dominican Republic, Brazil, Guatemala, Honduras, Nicaragua, Cameroon, and Uganda (27, 28).

### A method using mean intakes and EAR and UL as cut-points

Often relatively little information is available in low- and middle-income countries on the intake distribution of candidate foods for fortification. A special Excel-based tool has been developed (called the ‘Food Fortification Formulator’) to select the level of micronutrient addition to food requiring little input data by the user (29, 30). The user defines the intake goal in terms of filling the micronutrient gap of the target group as a percentage of the EAR value to be met by 95% of the population (29, 30). The user provides an estimate of the median usual consumption of the micronutrient of interest (from all food sources and supplements) by the target group. In addition, an estimated *per capita* consumption of the food vehicle is required corrected for the proportion of the general population consuming the food. The median consumption of the food vehicle by the target group is derived from the estimated *per capita* consumption of the food vehicle by adjusting for relative energy requirements by age and gender group (31). The 5th and 95th percentiles are estimated by applying an adjustment factor to the median. Other required estimates include the bioavailability of the micronutrient in the diet (low or moderate), and overages to compensate for losses of the micronutrient in the food prior to consumption (during storage, transportation, and cooking). The contribution of the fortified food to the total micronutrient intake of the target group can then be simulated at different fortification levels. The tool proposes a level of fortification that meets the defined proportion of the EAR of the target group at the 5th percentile. The tool also proposes a safe level of fortification that does not exceed the UL of the target group at the 95th percentile. This is done so by dividing the safe additional micronutrient intake between the UL and the usual intake of the micronutrient at the 95th percentile, by the consumed amount of the food vehicle at the 95th percentile. Apart from the UL, the costs or technological limitations of the added micronutrient can be another constraint when setting the level of fortification. The tool is relatively easy to use, and accounts for bioavailability and overages. A major drawback is that the intake medians and distributions in the target group rely on a number of assumptions. Moreover, since energy density rather than portion size determines food consumption, it was recently proposed to base calculations on the contribution of fortified foods to micronutrient intake in terms of energy (kcal/d) rather than amount (g/d) and express fortificant content in food per kcal instead of kg (32). The concept of fortifiable energy was also the basis for setting maximum amounts of safe food fortification in Europe (17, 33).

### The challenge of setting fortification levels using the EAR and UL as cut-points

Methods that use EAR and UL cut-points are based on the assumption that the micronutrient fortification level should contribute to reaching the EAR in the target population at the lowest intakes while not exceeding the UL at the highest intakes. However, shifting micronutrient intake so that the majority of the target population has intakes above the EAR and below the UL is not always feasible. The absence of adequate safety data for most micronutrients results in a separation between the UL and the EAR that can be quite narrow (20, 21). As consequence, intakes of vitamin A, calcium, copper, fluoride, iodine, iron, manganese, and zinc have a UL that is close to the EAR and usual intake and have a potential of exceeding the UL (34). Distributions of micronutrient intakes tend to be skewed. In particular, vitamin A has a wide distribution of intakes with a long right tail indicating that some individuals in the population may have very high intakes relative to others. As consequence, a large proportion may have intakes below the EAR while another proportion is close to or even exceeds the UL. Young children in particular are at risk of consuming micronutrient intakes below their EAR as they consume smaller food quantities but proportionally need more micronutrients per body weight than adults to meet their requirements. Moreover, they can also be at risk of exceeding their UL as the UL for young children has often been set conservatively to protect this sensitive age group.

This can be exemplified by vitamin A consumption data reported for India (35). The data indicate that the median usual intake of vitamin A by children aged 1–3 years of 61 µg Retinol Equivalent (RE)/d was far below their EAR of 286 RE µg/d. In contrast, their intakes at the 95th percentile were likely to exceed the UL of 600 µg/d given a mean intake of 151 µg RE/d, a SD of 308 µg RE/d, and a lognormal intake distribution (35). Another example of micronutrients likely to surpass both the EAR and UL is given by zinc intake data collected in three regions of Uganda (36). The data point out that 50% of children 6–23 months of age had usual zinc intakes with assumed low bioavailability below the EAR of 3.4 mg/d. However, in two of the three regions, usual zinc intakes of children in the 95th percentile exceeded the UL of 7.0 mg/d (36).

When planning to increase micronutrient intakes, intake distributions between the boundaries of the EAR and UL cannot always be achieved for some micronutrients. This scenario poses a dilemma for policy makers who need to decide on the acceptability of not addressing intakes below the EAR or allowing the UL to be exceeded. Not allowing intakes to exceed the UL by high-end users may result in fortification levels that only partially meet the target EAR.

To illustrate this dilemma, an example of sugar as a food vehicle for vitamin A fortification can be examined using the Food Fortification Formulator (29). Assumed is a national *per capita* sugar supply of 55 g/d [equivalent to the global average sugar supply in 2011 (37)], and a *per capita* sugar consumption of 61 g/d after correcting for 10% of the population estimated not to consume sugar. The tool assumes the 55 g/d *per capita* consumption to represent adult male consumption and calculates a median sugar consumption of 23 g/d by children 1–3 year of age by adjusting for lower energy intake by children (1,100 kcal/d) compared to adult males (3,000 kcal/d). The model provides sugar intake estimates of 7.5, 23, and 45 g/d at the 5th, 50th, and 95th percentiles, respectively. In this example, a usual vitamin A intake of 150 µg RE/d is assumed. Based on an average sugar consumption pattern, the tool suggests a fortification level of 15 mg vitamin A per kg sugar. The tool also provides a safe vitamin A fortification level of 10 mg/kg sugar by dividing the safe intake margin (450 µg/d before reaching the UL of 600 µg/d) by the 95th percentile sugar intake (45 g/d). The tool considers 28% loss of vitamin A prior to consumption (during production, storage, distribution, and cooking). Selecting a conservative vitamin A level of 10 mg/kg sugar would consequently represent a vitamin A level of 7.2 mg/kg sugar at consumption. The 1–3 year olds in the 5th percentile consuming 7.5 g/d of sugar would consume only 54 µg/d of vitamin A, that is, 19% of their EAR of 286 µg/d. Therefore, when choosing an efficacious level, the 95th percentile may exceed their UL, whereas choosing a conservative level, a large proportion of children may be at risk of inadequacy. This choice requires better understanding of the actual risks below the EAR and above the UL.

### Reaching the target population while avoiding excessive intakes in other groups

If the target population at risk of micronutrient deficiency is well-defined and reachable, targeted micronutrient interventions are the approach of choice. However, when the micronutrient deficiency is widespread across the entire population, mass food fortification is the preferred approach. When implementing mass fortification, the choice of the food vehicle is of key importance; the vehicle should be consumed in constant predictable amounts by the target population (9). If consumption of the fortified food is not evenly distributed either geographically or demographically, a wide distribution of intakes may result, which would be undesirable. For example, following the introduction of universal fortification of sugar with vitamin A in Guatemala, xerophthalmia in children aged 6 months to 10 years declined by 30% from the 1980s to the early 1990s and further declined by 70% with additional high-dose vitamin A supplementation in the late 1990s (38). However, sugar nowadays is mainly consumed by the urban Guatemalan population and less by the rural population, who are at greatest risk of vitamin A inadequacy (39). Moreover, the level of vitamin A consumption from sugar may nowadays exceed the UL of some groups at the 95th percentile (39, 40).

**Fig. 2:**
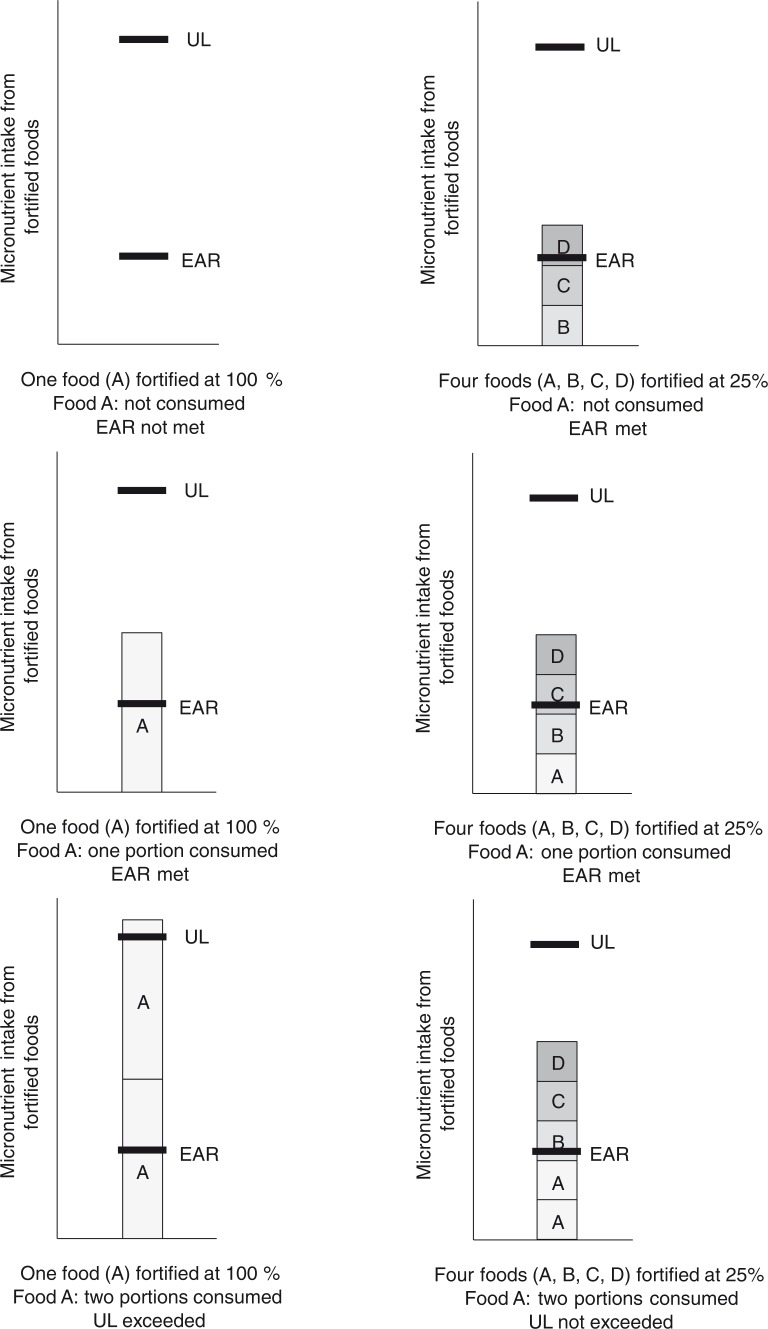
Individual micronutrient intakes from one food A fortified at 100% relative level (left) or micronutrient intakes from four foods A, B, C, and D fortified at 25% relative level (right). Scenarios include consumption of no fortified food A (top), one portion of fortified food A (middle), or two portions of fortified food A (bottom). Top: when no food A is consumed, the EAR would *not* be met with only food A fortified, but *would* be met with foods A, B, C, and D fortified even at 25% level. Bottom: when two portions of food A are consumed, the UL would be exceeded with only food A fortified, but the UL would *not* be exceeded with foods A, B, C, and D fortified.

Dividing the micronutrient fortificant amount across multiple food vehicles instead of a single vehicle can be a suitable approach to narrow the intake distribution from mass fortification; multiple food vehicles are more likely to reach different segments of the population and hence to reach the target population while being less likely to be all consumed by consumers with the highest intakes (Fig. 2).

## Tiered approach for risk–benefit assessment of foods

As previously stated, for some micronutrients and population groups, shifting micronutrient intakes between the boundaries of the EAR and UL may not be feasible and thus poses a dilemma for policy makers. By not allowing the UL to exceed when setting micronutrient levels will ensure safe intakes within the population but can leave a proportion of the population at risk of inadequacy, and *vice versa*. Nevertheless, adverse health implications for intakes below the EAR and above the UL may differ substantially in magnitude (19). For example, the first adverse effect that is observed at the high end of intake determining the UL may be non-severe and reversible and include a wide margin of safety whereas the first adverse effect observed at the low end of intake determining the EAR may be more severe or *vice versa*. Using the EAR and UL as cut-points may ensure that a population is at acceptable low risk of inadequate and excessive intake, respectively, but does not give information about the type and magnitude of the risks when these reference points are surpassed.

The use of large uncertainty factor values for young children, particularly for vitamin A, niacin, and zinc, has been subject to much debate as to whether they are limiting effective micronutrient interventions (21, 41, 42). Several agencies recognized that where the margins between requirements and UL are narrow, application of conventional methods of risk assessment, such as those using the established EAR and UL as cut-points, could result in recommended safe levels which would be below those that are essential (34, 43). Thus, when doubt exists as to the risk of excess intake versus the benefits of reducing inadequate intake by a micronutrient intervention, the traditional cut-point method may not provide sufficient information. In order to overcome these shortcomings, risk–benefit approaches have been developed that assist in addressing the question whether the health risks outweigh the health benefits or *vice versa* (44–46). By quantitatively expressing the adverse health impact related to inadequate and excessive micronutrient intakes and weighing them against each other helps in decision making (18). A number of European projects, including the ‘Best Practices in Risk–Benefit Analysis’ project, have evaluated how to best assess foods and food components (47, 48). Several committees, for instance the European Food Safety Authority (EFSA), make use of a tiered (stepwise) risk–benefit assessment approach to evaluate and manage potential changes in risks and benefits of dietary consumption patterns (49).

The risk–benefit approach is a tiered approach allowing for several ‘decision’ opportunities, depending on whether the available information is sufficient to address the initial risk–benefit question. For example, in the Benefit Risk Analysis for Foods (BRAFO) approach, risk–benefit assessment follows a four-step approach (50). Upon formulation of a risk–benefit question, in which at least two scenarios are defined, in the initial step, adverse health risks and benefits are identified in the different population groups without using health metrics. The question addressed is whether, at the relevant nutrient intake level, the health benefits clearly outweigh the health risks or *vice versa*. If not, then a second step is undertaken, requiring modeling of the relationships between micronutrient intake dose and adverse effect incidence at the two ends of the intake spectrum to estimate the effect size of increasing nutrient intakes (47). Biomarkers, clinical signs, or symptoms of inadequacy or toxicity may be used to establish intake dose–adverse effect response curves. In this respect, new and promising methods have been developed to address the relationship between intake and health-related status biomarkers that can be used for dietary planning (51). If in this second step risks and benefits of increasing the micronutrient intake still do not clearly outweigh each other, then the beneficial versus adverse effects are subsequently balanced using quantitative measures in a third step. If sufficient information is available, the health outcome is preferably expressed as change (gain or loss) in a composite health metric such as the Disability Adjusted Life Years (DALYs), or Quality Adjusted Life Years (QALYs). The DALY and QALY are a well-accepted public health measures describing burden of disease and are comprised of different dimensions of incidence, duration, severity/disability of the micronutrient intake-related adverse health conditions, and morbidity. Inputs required by the user include demographics (age, gender, and life expectancies), usual and target micronutrient intakes, dose–response curves for micronutrient-related adverse health effects, age of onset, duration, recovery probabilities, mortality probabilities, and weighted severity of the given micronutrient. The WHO has developed an Excel template that enables calculation of the health burden for different age and gender groups before and after micronutrient intervention in terms of DALYs (52). The QALIBRA project (Quality of Life Integrated Benefit and Risk Analysis) published a statistical software program that assists in modeling the probability of adverse health effects as function of micronutrient intakes, using QALYs or DALYs taking inter-individual variability and uncertainties into account by probabilistic modeling (53).

The step-wide risk–benefit approach has been applied to different food and nutrient intervention strategies in industrialized countries. By using the BRAFO approach, the net quantitative health impact of increasing folic acid intakes via flour fortification was assessed in the Dutch population (44). It was shown that flour fortification at the level of 140 µg/d of folic acid achieved the largest health benefit; the health loss resulting from masked vitamin B12-deficiency in terms of DALYs appeared negligible compared to the health gain resulting from prevented neural tube defects. Recently, an additional step in risk–benefit assessment was published that allows to find the scenario that provides the maximum net health gains using vitamin D intake as an example (54).

In developing countries, the risk–benefit approach could particularly be of interest to micronutrients with intakes below the EAR and close to UL or where multiple foods are fortified and varying consumption patterns exist within the population (such as in Guatemala or Cameroon where multiple foods are fortified with vitamin A with large variability of vitamin A intake within the population). When using a risk–benefit approach, data from health and demographic surveys are required on population demographics, and incidence rates of relevant diseases and mortality. Moreover, micronutrient intake distribution estimates are required to estimate risks of inadequate and excess micronutrient intakes. However, few countries have morbidity, mortality and food intake data available, even less have simulations of micronutrient intake distributions with programs like IMAPP. It remains to be seen whether risk–benefit simulations of food fortification scenarios, requiring even more inputs, will be performed. Nevertheless, improving data collection and methodology is an investment with a high return allowing authorities to make important public health decisions focusing on optimal public health perspectives in the targeted population.

## Tools to prioritize interventions based on cost-effectiveness

Despite the substantial micronutrient-related public health risks that exist, many countries may be hesitant to take a fortification approach for a number of reasons, such as loss of consumer choice, the health benefits of the micronutrients are not sufficiently proven, the UL may be exceeded, or there may be perceived health risks. Any uncertainty associated with initiating a micronutrient intervention can be addressed by expressing the effectiveness of the intervention in terms of a quantitative measure such as DALYs or QALYs gained or lost (13, 44, 55). Relating the cost of an intervention to the expected health gain can help to prioritize public health interventions. Nutrition interventions are generally highly cost-effective both in industrialized countries and developing countries (13, 56). Nevertheless, few cost-effectiveness assessments have integrated health losses related to excessive intakes of nutrients with an upper intake level. A recent assessment of mandatory folic acid fortification of bread in Australia showed that even when taking into account both potential beneficial and adverse health effects of folic acid, the intervention can be very cost-effective (57).

Another cost-benefit prioritizing tool is currently being developed which can assist policy makers in developing countries to make strategically prioritized investment decisions on, for example, micronutrient interventions based on their impact on disease reduction and costs (58). The public health benefit of micronutrient interventions is estimated in terms of percent reduction of the target population with inadequate intakes below the EAR. However, other risk aspects such as severity, duration, and incidence of the micronutrient deficiency are not taken into account. Information on micronutrient intake distribution in relation to the EAR, actual program coverage, and related costs is required. This allows the user to select the micronutrient intervention (combinations) that can achieve the largest percent reduction of intakes below the EAR at minimized costs and maximized coverage in the target population.

## Conclusion

Conventional methods to increase micronutrient intakes within a population make use of the intake distribution in relation to the reference values of the micronutrient. However, the goal to minimize the proportion of individuals below the EAR and above the UL can pose a dilemma: should a proportion of the population at risk of inadequacy be left unaddressed to avoid intakes above the UL or *vice versa*? Intakes below the EAR and above the UL do not represent the same risk in terms of magnitude of adverse health effects but those above the UL may include large uncertainties (18). A risk–benefit approach can support decision making about increasing micronutrient intakes by gaining insight into the magnitude of risks involved at the two ends of the intake spectrum and by making choices about their acceptability. Performing a risk–benefit assessment requires collection, compilation, and modeling of data on morbidity, mortality, and micronutrient intakes. Although this requires an investment in expertise and resources, risk–benefit approaches promise to guide optimum decision making in micronutrient programs with smaller uncertainties than those applied to the more gross nutrient reference values traditionally used in standard risk assessment.
